# Effectiveness of Interventions Based on Pain Neuroscience Education on Pain and Psychosocial Variables for Osteoarthritis: A Systematic Review

**DOI:** 10.3390/ijerph19052559

**Published:** 2022-02-23

**Authors:** Leidy Tatiana Ordoñez-Mora, Marco Antonio Morales-Osorio, Ilem D. Rosero

**Affiliations:** 1 Health and Movement Research Group, Physiotherapy Program, Faculty of Health, Universidad Santiago de Cali, Santiago de Cali 760033, Colombia; ilemdayana@gmail.com; 2 Grupo Internacional de Investigación Neuro-Conductual (GIINCO), Universidad de la Costa, Barranquilla 080002, Colombia; marco.morales.osorio@gmail.com; 3 Facultad de Salud, Carrera de Kinesiología, Universidad Santo Tomás, Arica 1000000, Chile; 4 Facultad de Ciencias de la Salud, Carrera de Kinesiología, Universidad Arturo Prat, Iquique 1100000, Chile

**Keywords:** osteoarthritis, chronic pain, physical therapy, neurology, education, catastrophizing

## Abstract

Osteoarthritis (OA) is the most common joint condition. It affects more than 300 million people worldwide, who suffer from pain and physical disability. Objective: To determine the results of cognitive educational interventions for pain management and psychosocial variables in adults with OA. Method: A systematic review was conducted based on searches in MEDLINE, OVID, LILACS, Scopus, PEDro, OTseeker, The Cochrane Library, EBSCO, and Google Scholar. The search strategy included the main terms neuroscience education and osteoarthritis, without any re-strictions with regard to dates or study type (PROSPERO register CRD42021222763). Results: We included four articles that implemented the intervention in 1–6 sessions, addressing concepts related to goal orientation and providing strategies for understanding pain. The results suggest that there is an improvement between the groups (PNE) when compared, but this cannot necessarily be attributed to pain neuroscience education (PNE), as small effect sizes for variables such as pain catastrophizing and kinesiophobia were observed. The response in the modulation of acute pain following the surgical procedure may produce a variation in the responses and this may be mediated by medications. Conclusion: The study revealed an improvement in favor of the groups managed with PNE, although more studies documenting the topic are warranted.

## 1. Introduction

Osteoarthritis (OA) is the most common joint condition. It affects more than 300 million people worldwide, who suffer from pain and physical disability [1]. Osteoarthritis (OA) is the most commonly observed joint condition in the adult population in any region of the world. Its prevalence varies according to geographical location, ethnic group, sex, age, and affected joint [2]. The Global Burden of Disease study in 2017 showed a prevalence of 3754.2 per 100,000 cases [1,2,3]. It affects 1 in 8 men and women in the US (about 27–31 million people) and about 302 million people worldwide [4]. The joints in the knees, hips, and hands are the most affected [4], and, reportedly, the joint with the greatest symptomatology is the knee joint. Globally, it is estimated that 250 million people have OA of the knee [5]. OA is a chronic debilitating disease of the mobile joints characterized by the deterioration of the articular cartilage, alteration of the subchondral bone, formation of osteophyte, narrowing of joint space, and inflammation of the synovial membrane [6].

According to different research studies, adults are more affected by this disease, which worsens with age. It has been reported that about one-third of all adults globally present radiological signs of OA and must deal with pain, which leads to a reduction in mobility that can result in disability and difficulty in maintaining independence [7]. Pain severity is associated with functional limitations, disability, and health-related quality of life (HRQoL), which is impaired in patients with this pathology. Functional limitations may be of particular relevance when OA affects the upper extremities [7]. One study reported an association between HRQoL, threshold, pain perception, and passive coping strategies with chronic pain (specifically withdrawing, worrying, and resting) in patients with OA [8].

Pharmacological options for symptom management include the use of oral corticosteroids, opioids and, in some cases, intra-articular corticosteroid injections [9,10]. This is in addition to implementing non-pharmacological approaches, such as education, exercise, and weight loss, which are the cornerstones of these treatments. Such treatments have proven to be effective, but they require changes in the patient’s behavior, which are difficult to obtain. Exercise and weight loss improve functionality and reduce pain [11,12,13]. Education enhances compliance with and adherence to exercise and weight loss programs, which improves their long-term benefits [14]. Other more traditional treatments, such as orthopedic manual therapy, the use of splints, and the physical agent modalities approach provide short-term benefits in reducing pain, and improving function and physical performance in patients with OA [15,16,17]. In turn, there is a tendency to generate an improvement in multicomponent interventions using adherence-related educational processes [18], consequently resulting in an improvement in pain, among the multiple benefits observed [13]. Modern pain neuroscience has improved the understanding of chronic musculoskeletal pain, including the role of central sensitization [19]. In the last decade, an educational model on pain biology and physiology has been recognized as a compelling approach toward chronic pain management [20,21,22]. This model refers to a variety of educational interventions [23] and has been outlined using the following terms: explanation of pain [24], therapeutic neuroscience education, and pain neuroscience education (PNE) [25].

PNE is increasingly used as part of physiotherapy treatment in patients with chronic pain. A thorough clinical biopsychosocial assessment is recommended prior to PNE to allow for an adequate explanation of pain neurophysiology and biopsychosocial interactions, and for this process to be patient-centered [26]. Therefore, new studies are needed that can evaluate these treatment pathways [27]. No systematic review or meta-analysis exists, although there are a number of studies investigating the effectiveness of cognitive educational interventions as an adjuvant therapy in patients with OA. This systematic review aims to determine the outcomes of cognitive educational interventions on pain and psychosocial variables in adults with OA.

## 2. Materials and Methods

This study followed the Cochrane Collaboration specifications for systematic reviews [28] and the criteria included in the PRISMA checklist [29]. The protocol was included in the international prospective register of systematic reviews (PROSPERO CRD42021222763).

### 2.1. Eligibility Criteria

The following research question (PICO) was established:

Population: Adult patients (18 years of age and older) with OA.

Intervention: Cognitive educational interventions (PNE, pain neurophysiology, pain therapeutic education, explanation of pain).

Comparison: Conventional therapy or treatment.

Results: Pain intensity, stress level, catastrophizing, kinesiophobia, disability, and quality of life.

Type of study: Randomized controlled clinical trials, experimental studies, quasi-experimental studies, and pilot studies that assessed the effects of the interventions and included outcome measures.

Exclusion criteria: Studies in which the intervention used did not correspond to neuroscience education or the interventional strategy referred to an area other than physiotherapy, and studies with cognitive behavioral therapy or education that did not include the neuroscience component were excluded.

### 2.2. Information Source and Search Strategy

The study was carried out by resorting to the following databases, digital bibliographic libraries, or search engines: MEDLINE, OVID, LILACS, Scopus, PEDro, OTseeker, The Cochrane Library (Cochrane Central Register of Controlled Trials), EBSCO, and Google Scholar. The search strategy included Neuroscience education and Osteoarthritis as the main terms. The complete strategy is described in the PROSPERO protocol and Appendix A: (Pain Education OR pain neurophysiology OR neuroscience education OR pain neuroscience education AND chronic pain AND osteoarthritis OR osteoarthritides); also see Appendix A. The search was not restricted to language or date of publication. The searches were conducted between 22 November 2020 and 31 July 2021.

### 2.3. Selection of Studies

Study selection began with a calibration process for the selection. Two researchers (LTO and MMO) blindly and independently initiated the filtering processes after searching the different databases. Each researcher produced a list of studies after analyzing the title and abstract of each article. The article was included when there was agreement between the reviewers and, in the case of discrepancies between them, a third reviewer (IDR) decided on the inclusion of the article. The reviewer was blinded to the answers given by each researcher. Eligibility criteria were applied to the full-text analysis in the final selection. Any disagreement between the authors regarding eligibility, quality, and data retrieved from the studies was resolved by consensus.

### 2.4. Information Gathering Process

Data extraction was performed independently using an Excel-generated format (LTO), which included the first author and year, research design, country, sample size, age, study type, pathological stage, time elapsed since diagnosis, study objective, presence of pharmacological treatment, description of the PNE-based intervention, description of the comparison intervention or additional interventions, scale used, and outcomes in terms of pain, stress level, catastrophizing, kinesiophobia, disability, quality of life, adherence, and adverse events reported, if any. These results were used to present the mean and standard deviations per reported outcome. MMO and IDR confirmed the accuracy of the information.

### 2.5. Quality Rating, Risk of Bias and Certainty of the Included Articles

The quality of the included studies was assessed blindly and independently using the MINORS scale [30]. This evaluates the presence of a clearly established objective, the inclusion of consecutive patients, collection of prospective data, analysis adapted to the design/results presented, reporting of data loss, and equivalence between groups. Subsequently, a decision was made to include a given study considering a score of 11 as the minimum criterion. For studies corresponding to clinical trials, the PEDro [31] scale was used, which evaluates randomization, allocation concealment, similarity of baseline characteristics, participant masking, therapist masking, assessor masking, outcome data on at least 85% of participants for at least one primary outcome, intention-to-treat analysis, and statistical comparisons between groups and their outcomes.

If possible, data analysis was performed using Software Review Manager (Version 5.3, London, UK). Data were extracted using the weighted mean difference between groups. Heterogeneity was measured with I2, considering values greater than 40%. Clinical heterogeneity was reviewed by analyzing the variability of the participants, the interventions, and the results. Certainty Analysis to generate this process used the tool Grade [32], which provides explicit criteria for rating the quality of evidence, including study design, risk of bias, imprecision, inconsistency, indirectness, and magnitude of effect, with the support of GRADEpro GDT [33].

## 3. Results

**Figure 1:**
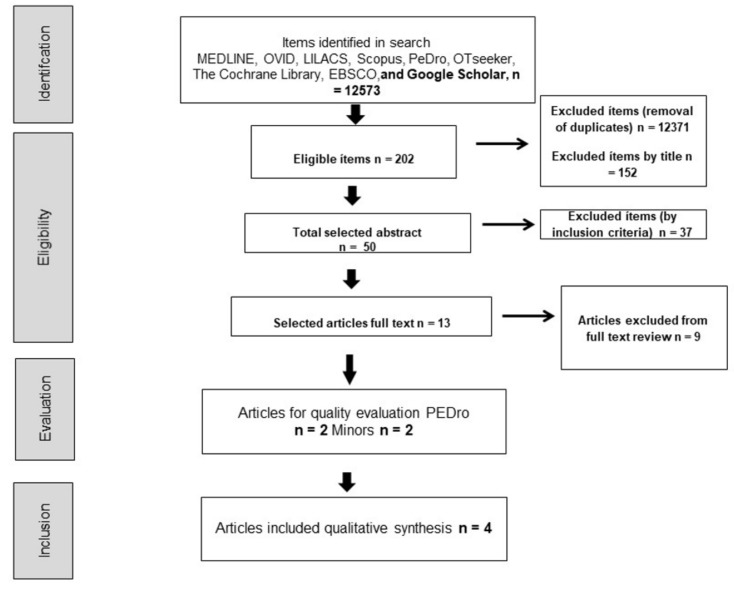
Search flowchart. Source: Own source.

Considering the systematic searches, a flowchart corresponding to the initial inclusion of articles and the application of filters was made. Starting with 12,573 titles eligible for selection, 202 unique records remained after the removal of duplicates. Finally, four articles were included in the qualitative synthesis of this study, as shown in Figure 1 and Table 1.

### 3.1. Description of the Intervention

The PNE intervention was performed by a physiotherapist. The duration of the sessions varied: 40–60 min in one study [34], 30 min in two studies [35,36], and 60 min (first session) and 30 min (remaining sessions) in one study [37].

Regarding the description of the intervention, the sessions addressed the development of the following aspects as the interventions were performed: 1. Purpose of neuroscience education, which included intervention objectives [34]; 2. Explanation of biological models and pathways [34,37]; 3. Pain and sleep, including pain distraction and inactivity; 4. Pain and lifestyle; 5. Self-care; 6. Decisions [34], predisposing factors to sensitization, the pain matrix, pain reconceptualization, surgical experiences, and knowledge transfer [37].

The sessions used prepared images to explain pain, ranging from the definition of an action potential to pain processing. Metaphorical material was also used, with examples making reference to the content [34,35,36,37], thereby enabling the possibility of creating PowerPoint presentations or using brochures [35]. Additionally, one of the studies used a different approach, i.e., book reading, to explain pain [24,37].

The results of the pharmacological treatment did not reveal any changes in the medication dosages of the groups with which they were compared, nor were any significant differences observed in the groups [34,35]. Two studies did not report on medications [34,35]. The adherence levels reported in the studies were 84% [34], 63% at 1-year follow-up, 100% [36], and 81% [37]. None of the studies reported adverse events resulting from the PNE intervention.

Description of Patients with OA

The studies reported on patients with radiologically proven OA (with diagnosis time of over 6 months) and candidates for total knee arthroplasty [34,37]. In the other studies, patients met the Western Ontario and McMaster Universities Osteoarthritis Index (WOMAC) diagnostic criteria and were candidates for total knee replacement [35,36].

### 3.2. Assessing the Quality of the Evidence

#### 3.2.1. MINORS Scale

**Figure 2:**
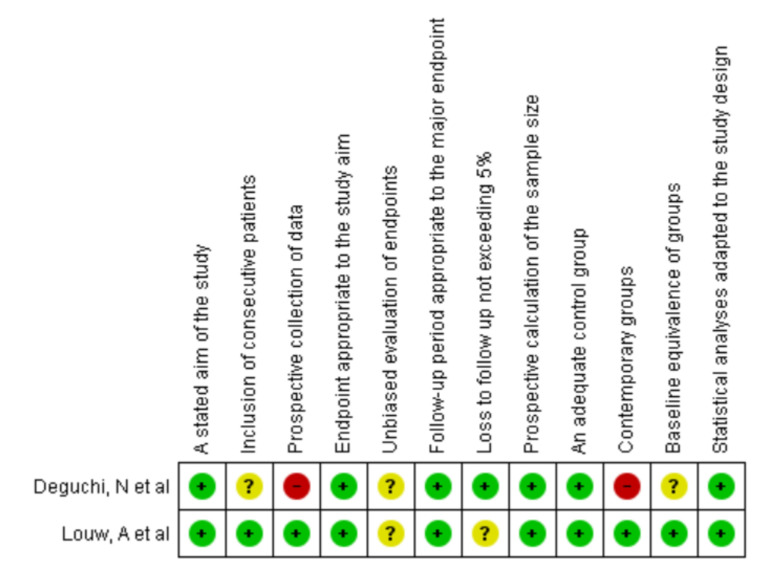
Risk of bias with the Minors scale. Source: Own source with Revman Manager 5.3.

The main reported methodological flaws were found in the prospective evaluation of the data, the equivalence and management of the groups, and the masked evaluation of the groups. However, due to the nature of the questionnaires used for pain study processes, many surveys were self-administered. See Figure 2.

#### 3.2.2. Pedro Scale

**Figure 3:**
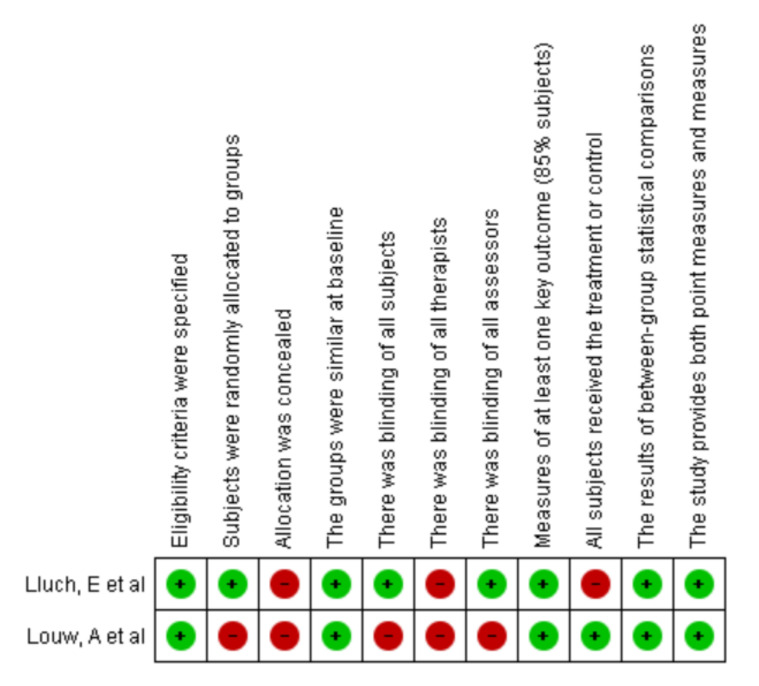
Risk of bias PEDro scale. Source: Own source with Revman Manager 5.3.

With regard to the evaluation of the clinical trials, there were shortcomings in terms of the masking in the Louw study [35] and in the allocation concealment. See Figure 3.

Table 2 shows the main results of the measurements pain, catastrophizing, kinesiophobia, disability, and quality of life found in the review. A meta-analysis of the data was not possible because of heterogeneity and a lack of unanimity in the interventions presented.

### 3.3. Pain Results

#### 3.3.1. PNE Compared with Another Intervention

A study added PNE to the conventional rehabilitation process. In this study, significant results were found with regard to the intensity of pain, both at rest and while walking, in favor of the group that included PNE when compared with the second group [34].

#### 3.3.2. PNE Compared with Other Educational Processes

A study compared PNE with traditional preoperative management. In this case, the two groups showed an improvement in pain modulation without significant differences between their results [35]. Conversely, no significant changes or differences between groups were found in a study that used PNE in combination with knee joint mobilization [37].

#### 3.3.3. PNE with No Comparison

One study evaluated the effect of PNE on a single group and reported no significant changes in pain reduction when compared to the baseline [36].

### 3.4. Catastrophizing Results

#### 3.4.1. PNE Compared with Another Intervention

For catastrophizing, significant results were reported, *p* = 0.036, in favor of the PNE group [34].

#### 3.4.2. PNE Compared with Other Educational Processes

No differences were reported in this variable in a comparison between the groups [34]. In another study, post-intervention changes were observed in favor of the PNE group and at 1 month after treatment when compared with the baseline, but this difference evened out at the 3-month follow-up [37].

#### 3.4.3. PNE with No Comparison

No significant differences were found in the baseline and post-intervention outcomes for this measure [36].

### 3.5. Kinesiophobia Results

#### 3.5.1. PNE Compared with Other Educational Processes

No differences were reported for this measure between the two groups, but a significant effect could be observed over time when compared with the baseline in the control and intervention groups [35].

Significant changes in this measure were reported 3 months postoperatively in favor of the PNE group compared with the control group [37].

#### 3.5.2. PNE with No Comparison

Significant changes compared with the baseline were reported for kinesiophobia [36].

### 3.6. Disability Results

#### PNE Compared with Other Educational Processes

There were no significant differences attributed to either group. There was improvement in this score at 1 month, but the difference leveled off over time [35]. The WOMAC score was lower for both groups 1 month after treatment when compared with the baseline (*p* < 0.01) [37].

### 3.7. Quality of Life Results

#### PNE Compared with Another Intervention

A statistically significant difference was found in favor of the PNE group regarding the quality of life measured using the Pain Self-Efficacy Questionnaire score [34].

### 3.8. Certainty Analysis

The certainty analysis is presented, each outcome measure evaluated was included and, due to the differences between the studies, these are addressed separately, and the degrees of evidence of the working group defined by GRADE were considered.

High certainty: We are very confident that the true effect is close to that of the effect estimate.

Moderate certainty: We are moderately confident in the effect estimate: the true effect is likely to be close to the effect estimate, but there is a possibility that it is substantially different.

Low certainty: our confidence in the effect estimate is limited: the true effect may be substantially different from the effect estimate.

**Figure 4:**
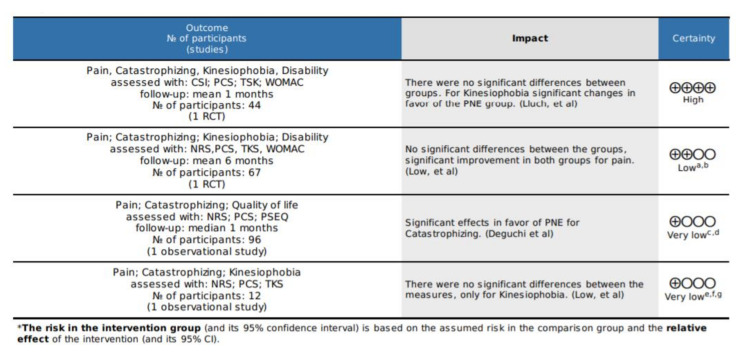
Certainty analysis with GRADE. Conventions: ^a^ One of the studies had no concealment or randomization and blinding of assessment. ^b^ Media and DS cannot be accessed, which makes it impossible to generate the meta-analysis. ^c^ There is no blinding of the evaluations, the collection of the patients is not clear. ^d^ The data differ from those reported with this scale in other studies. ^e^ there is no blinding of the evaluations, no second group was generated. ^f^ The measurements are generated in ranges. ^g^ The intervals are wide. Source: Own source with GRADEpro GDT.

Very low certainty: We have very low confidence in the effect estimate: the true effect is likely to be substantially different from the effect estimate. See Figure 4.

### 3.9. Characteristics of the Excluded Studies

The excluded studies were those that did not report data on the specific intervention of PNE or, when generating the process, another educational concept other than PNE was mentioned as conventional education without mentioning the neuroscience component. Similarly, studies that addressed a protocol, but excluded intervention without presentation of published results, were not considered. See Table 3.

## 4. Discussion

Four studies were included that involved the effects of PNE on patients with OA, in which a reduction in pain was found for the groups whose intervention included PNE, which may be due to pharmacological modulation without dismissing the effect of the educational intervention. Significance was found for variables such as kinesiophobia for the groups with PNE. There is a tendency to improve aspects such as quality of life. However, it was not a measure included in all the studies. There were no changes in disability level and the measure of stress level initially considered in the protocol was not addressed in any of the studies.

The process of neuroscience education aims to transfer knowledge to patients, allowing them to understand their pain and establish coping strategies, thereby decreasing erroneous beliefs and challenges associated with pain [47]. The results of this review suggest that there is an improvement when comparing the treatment groups. However, this cannot be attributed solely to PNE because small effect sizes were found in most studies for the main variables, such as pain catastrophizing and kinesiophobia [34,37]. This contrasts with the results reported by Louw et al. [48], who concluded that, in patients with chronic pain, there is compelling evidence that an educational strategy addressing the neurophysiology and neurobiology of pain can have a positive effect on pain, disability, catastrophizing, and physical performance. In turn, the response in the modulation of acute pain following the surgical process can produce variations in responses that can be mediated by medications (ranging from opioids to NSAIDs) and the therapeutic process [49].

Regarding pain intensity, no significant differences were found between the groups. Another systematic review that included different pathologies with chronic musculoskeletal pain found that PNE has a small-to-moderate effect on pain intensity with greater long-term effects for this variable. This could be explained by the fact that 11 of the 18 studies focused mostly on low back pain, the area most extensively researched with regard to PNE [50]. Regarding the results reported by Louw et al. [36], the intervention used was group-based, with a duration of 30 min, which may not be sufficient to estimate an actual change in the patient. However, no differences were found in the level of disability [35,37], which is consistent with the findings of another study in which the interindividual difference in the change in disability in response to PNE (7.36/100) did not meet the criterion for clinical significance (10/100). Therefore, there is insufficient evidence for PNE response estimation [51].

Among our recommendations, we suggest including preoperative work and reports regarding medication prescriptions. A previous study on lumbar radiculopathy included these variables, in addition to the cost estimation [52]. Louw also added this variable and reported no reduction in expenses or medication consumption with regard to knee arthroplasty [35]. This may be explained by the differences in the costs of post-surgical processes between spine and knee.

The fact that Lluch’s study did not include a control group for the comparison of the two interventions impedes establishing the adequacy of the interventions when compared with not receiving any education [37]. Limitations were found in all studies regarding the sample sizes, the adherence reported in the studies, which may result in the introduction of another type of conclusion in the analysis given by the protocol, and the heterogeneity in catastrophizing events, which can lead to variability. It is suggested that a report be fashioned based on the type of pain, which enables subgroup analysis, thereby allowing the establishment of the profile on which work can be conducted. Regarding the discrepancies with the originally published protocol of Prospero, the inclusion of only clinical trials had been considered, in addition to limiting the searches to this type of study; but due to the lack of literature, it had to be extended to observational studies, which allowed the inclusion of two more studies within the results.

During the execution of this study, the meta-analysis was a limiting factor, since it could not be unified or a sum of effects could not be generated. This is due to the fact that the interventions did not have the same dosing as each other and there were only four studies. Regarding the implications for practice, the inclusion of educational programs within the care processes in patients with OA is suggested. Thus, it is recommended that the authors of intervention studies standardize the evaluation measures in the presentation of the results and opt for the use of widely disseminated instruments used in other studies of the same type. This will allow an estimate of the size of the effect to be made.

## 5. Conclusions

Non-pharmacological and educational interventions should be carried out within the interventional processes in patients with pain. The findings revealed an improvement in the groups managed with PNE, finding a small effect in favor of the interventions for variables such as kinesiophobia, with no changes observed in the other variables evaluated. However, this may be due to pharmacological modulation, so further studies are warranted to make a recommendation regarding this intervention.

## Figures and Tables

**Table 1:** Characterization of the studies.

Author	Year	Population and Mean Age	Intervention	Control	Pharmacological Treatment	Scales Used	Results
Deguchi, N et al. [34]	2019	IG: 67 (67 years); CG: 52 (63.7 years)	Rehabilitation (same as the control group) + 6 PNE sessions were carried out by a physiotherapist, with each session lasting 60 min	Rehabilitation only (weight bearing as tolerated, 6 times a week in 40- to 60- minute sessions)	NSAIDs 3 times a day postoperatively, tapered off at 3 weeks postoperatively	NRS, PCS, PSEQ	No significant effects were found in the comparison items between groups, except catastrophizing.
Louw, A et al. [35]	2019	IG: 49 (74.1 ± 9.5) 31 analyzed; CG: 54 (69.6 ± 10.6) 36 analyzed	PNE	Traditional preoperative educational program	Opioid treatment according to the determined regulation	NRS, PCS, Tampa scale, WOMAC	No differences could be found between the groups.
Louw, A et al. [36]	2018	Patients = 12 [10 women + 2 men] (68.6 ± 8.7 years)	PNE	The second group was not managed	Not specified	NRS, PCS, Tampa Scale	There were changes in favor of the PNE group.
Lluch et al. [37]	2017	IG patients: 27 (72.8 ± 5.6); CG patients: 27 (67.7 ± 7.8)	PNE + knee joint mobilization	Biomedical education + Knee joint mobilization	Not specified	CSI, PCS, Tampa Scale, WOMAC	Changes were found regarding kinesiophobia and catastrophizing for PNE.

Abbreviations: IG (Intervention Group), CG (Control Group), PNE (pain neuroscience education), NSAIDs (non-steroidal anti-inflammatory drugs), (NRS (Numerical Rating Scale), PCS (Pain Catastrophizing Scale), PSEQ (Pain Self-Efficacy Questionnaire Score), Tampa (Scale of Kinesiophobia), WOMAC (Western Ontario and McMaster Universities Osteoarthritis Index), CSI (Central Sensitization Inventory).

**Table 2:** Results of the studies for pain, catastrophizing, kinesiophobia, disability, quality of life.

Author	Scale	Pain	Scale	Catastrophizing	Scale	Kinesiophobia	Scale	Disability	Scale	Quality of Life
Deguchi, N et al. [34]	NRS	Without significant changes in the groups IG: Pain at rest pre 2.0 (2.6) post 1.1 (1.3) CG: pain at rest pre 1.9 (2.3) post 0.8 (1.2)	PCS	Significant effects in favor of PNE IG: pre 30.3 (6.5) post 16.9 (9.7) CG: pre 30.8 (7.7) post 20.7 (8.4)					PSEQ	No significant changes between the groups IG: pre 37.6 (10.6) post 43.4 (10.2) CG: pre 36.3 (11.1) post 38.7 (12.8)
Louw, A et al. [35]	NRS	Significant improvement in both groups. There was a difference attributable to time (*p* < 0.001) with improvements in all patients.	PCS	No significant differences between the groups F (3192) = 0.209, *p* = 0.819, power = 0.083. (*p* = 0.075), yes, difference in time (*p* < 0.001)	Tampa Scale of Kinesiophobia	There were no significant differences between the groups F (3192) = 1.402, *p* = 0.245, power = 0.358 (*p* = 0.247)	WOMAC	There were no significant differences in the groups F (3195) = 1.501, *p* = 0.222, power = 0.355		
Louw, A et al. [36]	NRS	There were no significant differences between the pre- and post-intervention measures. pre PNE 5.0 IQR = 2.3–6.8 Range (0.0–8.0) post PNE 3.5 IQR = 1.0–5.0 Range (0.0–7.0) *p* = 0.119	PCS	There were no significant differences between the measures. pre 3.3–27.8 (1.0–51.0) post 7.0 IQR = 3.3–15.8 Range (0.0–36.0) *p* = 0.081	Tampa Scale of Kinesiophobia	A difference in favor of the PNE post intervention. pre 42.0 IQR = 38.5–44.0 Range (31.0–54.0) post 39.0 IQR = 36.0–42.5 Range (31.0–46.0) *p* = 0.036				
Lluch E, et al. [37]	CSI	There were no significant differences between the groups. IG: pre 37.6 ± 17.2 post 30.3 ± 10.2 CG: pre 38.3 ± 15.6 post: 38.1 ± 15.7	PCS	There were no significant differences between IG groups: Pre 22.6 ± 11.5 post 12.5 ± 10.3 CG: pre 25.9 ± 13.6 post 24.5 ± 13.6	Tampa Scale of Kinesiophobia	Significant changes in favor of the PNE IG were reported pre 34.3 ± 7 post 25.9 ± 5.9 CG: pre 33.7 ± 5.6 post 33.6 ± 6.7	WOMAC	There were no significant differences between the groups IG: pre 52.4 ± 14.6 post 41.4 ± 13.7 CG: pre 52.1 ± 18.4 post 50.1 ± 18.5		

Abbreviations: IG (Intervention Group), CG (Control Group), PNE (pain neuroscience education), NRS (Numerical Rating Scale), PCS (Pain Catastrophizing Scale), PSEQ (Pain Self-Efficacy Questionnaire Score), Tampa (Scale of Kinesiophobia), WOMAC (Western Ontario and McMaster Universities Osteoarthritis Index), CSI (Central Sensitization Inventory). Source: Own source

**Table 3:** Excluded studies.

Autor	Year	Type of Study	Reasons for Exclusion
Wang, L et al. [38]	2021	Clinical trial	It is a protocol, mention another educational technique
Larsen, J et al. [39]	2020	Clinical trial	It is a protocol
Lawford, B et al. [40]	2018	Clinical trial	it does not mention the specific technique
Saw MM et al. [41]	2016	Clinical trial	mention another educational technique
Bennell Kl et al. [42]	2016	Clinical trial	mention another educational technique
Fernandes L et al. [43]	2010	Clinical trial	mention another educational technique
Bezalel T et al. [44]	2010	Clinical trial	mention another educational technique
Baird CL et al. [45]	2004	Clinical trial	mention another educational technique
Ettinger WH et al. [46]	1997	Clinical trial	mention another educational technique

Source: Own source.

## Data Availability

Not applicable.

## Appendix A

Search strategies for the different databases

1. Pain Education
2. Pain Neuroscience
3. Pain Neuroscience education
4. Neurophysiology education
5. Therapeutics Neuroscience Education
6. Chronic Pain
7. Osteoarthritis
8. MH Chronic Pain
9. MH Osteoarthritis
10. 1 AND 6 AND 7
11. 1 AND 6 OR 8 AND 7
12. 2 AND 7
13. 3 AND 7 OR 9
14. 4 AND 7
15. 5 AND 7

MH = Medical Topic Headings, TX = Words
